# Surgical lobectomy of pulmonary arteriovenous malformations in a patient with presentations regarded as sequela of tuberculosis: a case report

**DOI:** 10.1186/s13019-020-01319-4

**Published:** 2020-10-02

**Authors:** Peng Teng, Weidong Li, Yiming Ni

**Affiliations:** Department of Cardiovascular Surgery, the First Affiliated Hospital, College of Medicine, Zhejiang University, 79#, Qingchun Road, Hangzhou, 310000 Zhejiang China

**Keywords:** Pulmonary arteriovenous malformation, Polycythemia, Cyanosis, Digital clubbing

## Abstract

**Background:**

Pulmonary arteriovenous malformations are uncommon conditions of abnormal communications between pulmonary arteries and veins, which are most commonly congenital in nature. Although such condition is not extremely rare, it is a challenge to the differential diagnosis of pulmonary problems such as hypoxemia and pulmonary lesions.

**Case presentation:**

We report a meaningful case of a 23-year-old male presented with elevated hemoglobin (23.0 g/dl) on admission. Physical examination revealed cyanosis, digital clubbing and low oxygen saturation on room air. The patient was initially diagnosed as polycythemia vera while the subsequent result of bone marrow aspiration was negative. During further assessment, pulmonary arteriovenous malformations were detected by CT pulmonary angiography. Lobectomy was successfully performed with significant increase in oxygen saturation from 86 to 98%. The hemoglobin decreased to almost normal level of 14.9 g/dl 3 months after surgery and the patient had been followed up for nearly 5 years.

**Conclusions:**

Pulmonary arteriovenous malformations should be suspected in patients with central cyanosis, digital clubbing, polycythemia, pulmonary lesion and without cardiac malformations. Embolization or surgery is strongly recommended to reduce the risks caused by pulmonary arteriovenous malformations.

## Introduction

Pulmonary arteriovenous malformations (PAVMs) represent an uncommon disease with a latest estimated incidence of approximately 1 in 2630 in population scanned by chest CT [1]. It was first described by Churton in 1897 [2]. Owing to the lack of intervening capillary bed, the patients with PAVMs have the predisposition to complications like hypoxemia, hemoptysis, ischemic stroke and cerebral abscess. Treatment is recommended to prevent later complications by embolization or surgical lobectomy. Herein, we report a 23-year-old male who presented with elevated hemoglobin which was initially treated as polycythemia vera on admission. His clubbed fingers and pulmonary lesion were misinterpreted as the sequela of previous tuberculosis (TB). Our case showed great learning value of differential diagnosis in patients with PAVMs. Furthermore, a brief literature review of PAVMs was performed.

## Case report

**Fig. 1:**
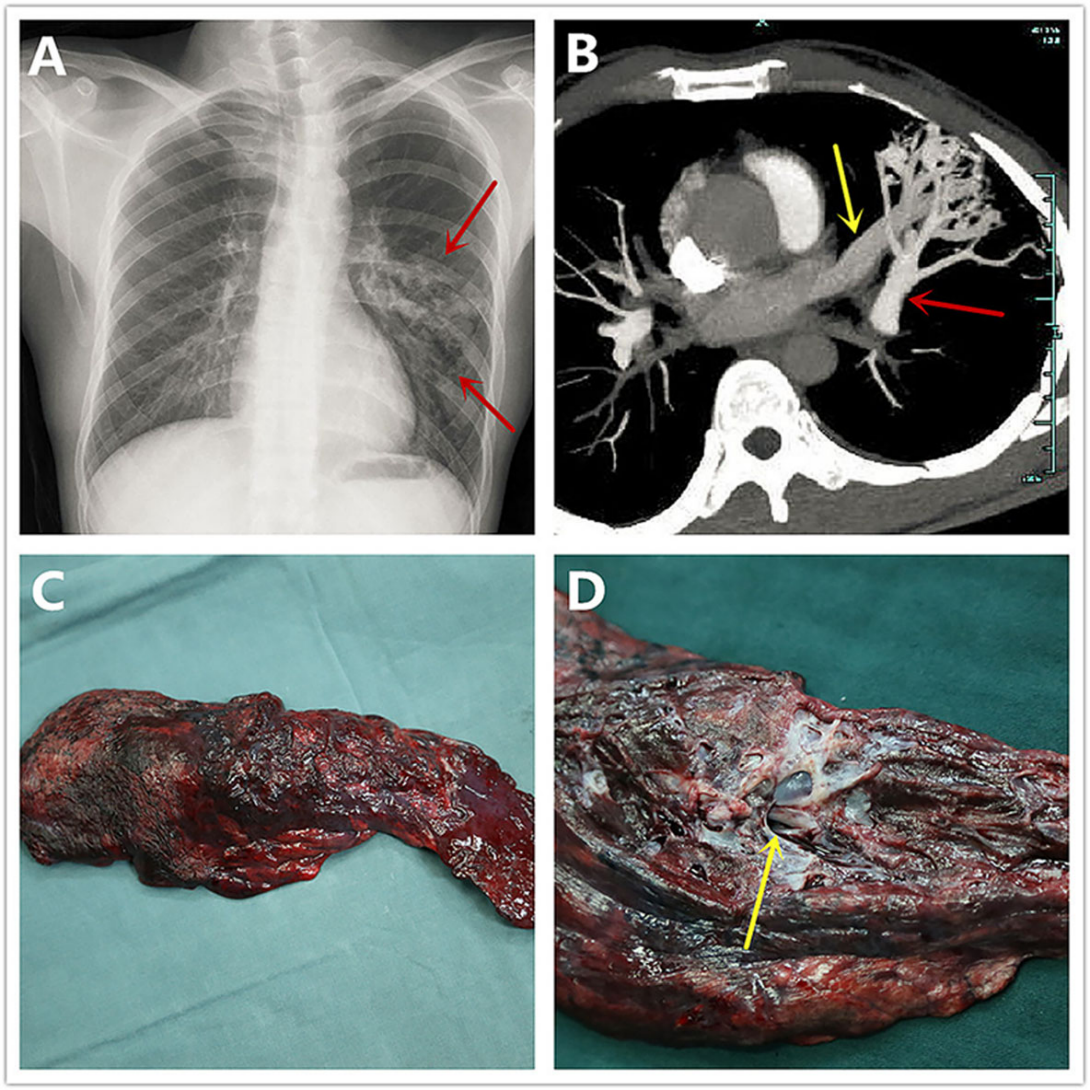
**a** Chest roentgenogram detected the lesions (in red arrows) in the left upper lobe which were initially considered as residual TB lesions. **b** CT pulmonary angiography showed abnormal communications between the left superior pulmonary vein (in yellow arrow) and left superior pulmonary artery (in red arrow). **c, d** Surgical specimen (excised left upper lobe) showed the diffusely tortuous vessels all over the lobe and the vascular cavities were extremely dilated (in yellow arrow). TB: tuberculosis; CT: computed tomography

A 23-year-old male was admitted to the department of hematology because of elevated hemoglobin during routine check-up. Physical examination revealed digital clubbing, cyanosis of oral mucosa and extremities. Remarkable signs of laboratory work up included decreased O_2_ saturation on room air of about 85% and elevated hemoglobin of about 23.0 g/dl. Chest roentgenogram suggested remote infectious lesion in the left upper lobe (Fig. 1a) and echocardiography showed no cardiac malformation. Additionally, the patient reported history of TB in his childhood and had been treated with standard anti-TB therapy for almost 1 year. Family members reported no history of repeated nosebleed, anemia or similar features mentioned above. The patient had been informed by his doctor that his clubbed fingers were sequela of chronic hypoxia caused by TB and the left pulmonary lesion was residual TB lesion after standard treatment. Bone marrow examination demonstrated non-specific erythroid hyperplasia. The initial diagnosis made by hematologist was polycythemia vera and hydroxyurea was applied. After several courses of treatment, the patient’s hemoglobin still kept high (22.5 g/dl) without obvious change and cardiothoracic consultation was scheduled. Considering the digital clubbing, cyanosis of oral mucosa and extremities, elevated hemoglobin, low O_2_ saturation and left pulmonary lesion, PAVMs were suspected. CT pulmonary angiography showed abnormal communications between the left upper pulmonary arteries and veins and PAVMs of left lingual lobe was confirmed (Fig. 1b). No extrapulmonary vascular malformations were detected.

Since PAVMs were diffuse but restricted to left upper lobe of lung, complete feeding arteries embolization was difficult and the patient finally received video assisted thoracoscopic left upper lobectomy. Intraoperatively, diffusely dilated and angiomatous vessels with hemorrhagic tendency were detected on the surface of left upper lobe (Fig. 1c, d). The left superior pulmonary vein, left upper lobe bronchus and left superior pulmonary artery were resected by endo-stapler, respectively. After left upper lobectomy, the O_2_ saturation immediately increased to 98%.

The histopathological examination confirmed the diagnosis of PAVMs. The patient was discharged home on the 5th postoperative day with hemoglobin level of 19.2 g/dl. Three months after surgery, the symptom of cyanosis relieved and hemoglobin was down to almost normal level of 14.9 g/dl. So far, the patient has been followed up for about 5 years without occurrence of cyanosis, dyspnea, decreased O_2_ saturation or increased hemoglobin level.

## Discussion

PAVMs are defined as abnormal communications between pulmonary arteries and veins without intervening capillary bed. Its prevalence is approximately 1 in 2630 in population scanned by chest CT with a slight predilection for female [1, 3]. PAVMs can be sporadic, multiple or even diffuse lesions as well as uni- or bi-lateral involved. PAVMs often have lower lobe predominance. Histopathologically, PAVMs manifest as tortuous or direct aneurysmal connection developing between arteries and veins without intervening capillary bed, which led to the loss of “filter capacity”.

The etiology of PAVMs could be congenital or acquired. The majority of PAVMs are associated with autosomal dominant disorder called hereditary hemorrhagic telangiectasia (Osler-Weber-Rendu syndrome) [4]. Acquired PAVMs are usually secondary to liver cirrhosis, infections, metastatic carcinomas, chest trauma and iatrogenic procedures [3]. Patients with PAVMs are usually asymptomatic, especially in children. The common pulmonary symptoms include cyanosis of oral mucosa and extremities, digital clubbing, hemoptysis and chest pain. Extrapulmonary manifestations are polycythemia, cerebral abscess, stroke or transient ischemic attack, epistaxis and so on [5]. In our patient, the hypoxemia as well as polycythemia were consequences of right-to-left shunt caused by PAVMs and were responsible for further presentations like digital clubbing and cyanosis.

In our case, the patient was initially treated as polycythemia vera. There might be several reasons why the other clinical features were neglected. First, the history of TB made the doctors regard the pulmonary lesion as residual TB lesion. Second, TB is able to cause cyanosis and digital clubbing when severe hypoxemia occurs, which could explain the patient’s cyanosis and clubbed fingers since he was a child. We should draw lessons from this case that PAVMs should be suspected when the following combination of clinical manifestations coexists: central cyanosis, digital clubbing, hypoxemia, hemoptysis, elevated hemoglobin, pulmonary lesions on chest roentgenogram or CT, history of cerebral abscess and so on.

Echocardiography findings of PAVMs might suggest increased left heart volume overload. Chest roentgenogram could detect abnormal lesions in PAVMs region but with low specificity. CT pulmonary angiography is of great diagnostic value. Pulmonary angiography is regarded as the gold standard for diagnosis, especially when a therapeutic intervention is planned. In patients who are allergic to iodinated contrast, contrast-enhanced transthoracic echocardiography is highly recommended to be used as a diagnostic method, in which early appearance of microbubbles in the left atrium strongly suggests the existence of PAVMs [3].

The natural history of PAVMs is relatively predictable because of its tendency to increase in size. If untreated, the mortality rate of untreated symptomatic patients ranges from 4 to 22% and even up to 40% in severe cases [6]. Spontaneous regression has rarely been reported.

There is consensus about early intervention in patients with PAVMs to prevent later complications like systemic embolization, pulmonary hemorrhage, ischemic stroke, cerebral abscess, congestive heart failure and so on. Treatment strategy of PAVMs should be made by taking multiple factors such as size, number, location and complications into consideration. Embolization is recommended as first-line treatment for PAVMs [7] while surgical lobectomy is recommended when PAVMs are diffuse, large and restricted to one lobe [8].

## Conclusion

We experienced a meaningful case with PAVMs which was initially treated as TB and polycythemia vera. We emphasize the importance of maintaining clinical suspicion of PAVMs in patients with features of central cyanosis, digital clubbing, hypoxemia, hemoptysis, elevated hemoglobin, pulmonary lesions on chest roentgenogram or CT, history of cerebral abscess and so on. Surgical lobectomy is suitable for PAVMs which are diffuse, large and restricted to one lobe.

## Data Availability

Please contact author for data requests.

## Abbreviations

PAVM: Pulmonary arteriovenous malformation
CT: Computed tomography
TB: Tuberculosis
